# Prenatal diagnosis of fetuses with ultrasound soft markers

**DOI:** 10.1186/s12884-025-08238-z

**Published:** 2025-11-06

**Authors:** Qianzhu Jiang, Chang Tan, Lin Yuan, Aziz Ur Rehman Aziz, Haihua Yu, Xiliang Wang

**Affiliations:** 1 Genetic Metabolism Medical Center, Dalian Women and Children′s Medical Center (Group), 21 Baishan Road, Dalian, Liaoning 116000 China; 2 Department of Reproductive Medicine, General Hospital of Northern Theater Command, 83 Wenhua Road, Shenyang, Liaoning 110000 China; 3 Key Laboratory for Early Diagnosis and Biotherapy of Malignant Tumors in Children and Women, Dalian Women and Children′s Medical Center (Group), 154 Zhongshan Road, Dalian, Liaoning 116000 China

**Keywords:** Ultrasound soft markers, Prenatal diagnosis, Chromosome microarray analysis, Chromosome karyotype analysis

## Abstract

**Objective:**

This study aims to evaluate the association between ultrasound soft markers and fetal chromosomal abnormalities and to compare the diagnostic efficacy of karyotype analysis versus chromosomal microarray analysis (CMA) for prenatal testing strategy optimization.

**Materials and methods:**

A retrospective review was conducted on 622 cases receiving prenatal diagnosis for abnormal ultrasound soft markers at our center over three years. All cases underwent chromosomal karyotype analysis and CMA testing. The differences between the results of these two tests, as well as the correlation between genetic testing results and abnormal ultrasound soft markers, were analyzed. Additionally, the pregnancy outcomes and postnatal phenotypes of all cases were monitored.

**Results:**

The overall prevalence of chromosomal abnormalities was 11.41% (71/622). Echogenic intracardiac focus (*P* = 0.012) and multiple soft markers (*P* < 0.001) exhibited a higher correlation with chromosomal abnormalities, with the latter showing a particularly strong association with aneuploidy (*P* < 0.001). Karyotype analysis identified 63 chromosomal abnormalities, while CMA detected 65, with discordant results observed in 18 cases. Among the cases with chromosomal abnormalities, 11 resulted in live births, and follow-up at ages 3–5 revealed no abnormal phenotypes.

**Conclusion:**

Prenatal genetic diagnosis is strongly recommended for pregnant women presenting with ultrasound soft markers, particularly multiple markers. Concurrent CMA and karyotype analysis are advocated to minimize the risk of missing pathogenic variants.

**Supplementary Information:**

The online version contains supplementary material available at 10.1186/s12884-025-08238-z.

## Introduction

The soft markers of the fetus are nonspecific, slight, and often temporary variations in fetal structure, including increased nuchal translucency (NT), absent nasal bone, choroid plexus cyst, short femur, mild ventriculomegaly, tricuspid regurgitation, aberrant right subclavian artery, echogenic bowel [1]. The incidence of isolated soft markers in normal fetuses is about 10% [2]. These markers often appear during a particular stage of fetal growth and development, and commonly resolve in the third trimester of pregnancy, but they may be associated with chromosomal abnormalities [3, 4].

Amniocentesis stands as the principal modality for prenatal genetic diagnosis, with a decades-long trajectory of clinical implementation. Although existing studies have demonstrated that this invasive procedure does not significantly increase the risk of pregnancy loss before 24 weeks of gestation, its clinical adoption remains constrained by procedural invasiveness [5]. Previous studies have investigated the prenatal diagnosis of cases with abnormal ultrasound soft markers, the clinical decision regarding whether pregnant women presenting ultrasound soft markers should undergo invasive diagnostic genetic testing remains complex [6–8].

Multiple modalities are currently available for chromosomal abnormality detection. As the gold standard for prenatal diagnosis, karyotype analysis enables reliable detection of aneuploidies. Meanwhile, chromosomal microarray analysis (CMA) based on comparative genomic hybridization (CGH) arrays and single-nucleotide polymorphism (SNP) arrays demonstrate superior performance in detecting copy number variations (CNVs) and uniparental disomy (UPD) [6, 9]. However, the optimal diagnostic strategy for pregnant women presenting with abnormal ultrasound soft markers remains to be determined.

To provide further reference information, we have reviewed prenatal diagnosis cases from January 2019 to December 2021, focusing specifically on those with ultrasound soft markers for comprehensive analysis. Our primary objective is to investigate associations between ultrasound soft markers and fetal chromosomal abnormalities, and to compare the diagnostic performance of karyotype analysis versus CMA. The findings of this study will offer invaluable references for prenatal genetic counseling, guiding decisions on the necessity of prenatal diagnosis and the selection of appropriate methods in relevant cases.

## Methods

### Participants

The study retrospectively analyzed cases at the Dalian Women and Children’s Medical Center (Group) from January 2019 to December 2021, specifically focusing on pregnant women who underwent amniocentesis for genetic testing after fetal ultrasound examinations revealed the presence of soft markers. Notably, pregnant women with confirmed fetal structural abnormalities were excluded from this study.

### Ultrasound examination

In our center, the ultrasound soft markers were identified during the first-trimester (11 ~ 13 weeks + 6 days) and second-trimester (20 ~ 24 weeks) scans (GE Voluson E8; GE Voluson E10). All ultrasound examinations were performed in strict accordance with the standardized protocols established by the International Society of Ultrasound in Obstetrics and Gynecology (ISUOG) and the Ultrasound Branch of the Chinese Medical Association [10, 11]. The ultrasound soft markers analyzed included: increased NT (11w ~ 13w + 6, ≥ 3.0 mm), absent nasal bone (including unilateral and bilateral absence), choroid plexus cyst (> 2 mm), short femur length (≤ 10 percentile), mild ventriculomegaly (≥ 10 mm and < 13 mm), tricuspid regurgitation, aberrant right subclavian artery, echogenic bowel (echo intensity equal to or exceeding that of adjacent bone), single umbilical artery, echogenic intracardiac focus, reversed A-wave in the ductus venosus, umbilical cord cyst, persistent right umbilical vein and pyelic separation (≥ 4 mm). The ultrasound soft markers evaluated in the first trimester include increased NT, absent nasal bone, reversed A-wave in the ductus venosus, and single umbilical artery. The remaining markers were assessed during the second trimester.

### Genetic counseling

For cases with ultrasound soft marker anomalies detected in the first trimester, non-invasive prenatal testing (NIPT) or amniocentesis were recommended [12]. For soft marker anomalies identified in the second trimester, amniocentesis was primarily recommended. Amniocentesis was optimally performed between 18 and 24 weeks of gestation. Procedures outside this window required rigorous risk-benefit evaluation and multidisciplinary review, with emphasis on maternal-fetal safety and procedural feasibility. All participants must be duly informed about the potential for false-positive and false-negative outcomes in NIPT [13]. Additionally, participants were informed that amniocentesis carries potential complications including fetal loss, premature rupture of membranes, chorioamnionitis, fetal injury, and severe maternal complications, though the incidence rates of these adverse events are extremely low [5, 14]. For pregnant women with contraindications to invasive prenatal diagnosis, including threatened abortion, bleeding diathesis, active chronic pathogen infection, or Rh-negative blood type, amniocentesis is contraindicated. Instead, NIPT is suggested as an alternative [14]. All participants voluntarily chose whether to undergo amniocentesis after providing informed consent.

### Chromosome karyotype analysis

The procedure involves collecting 20mL of the amniotic fluid from each patient under the ultrasound guidance (GE Voluson E10). For each sample, a minimum of 20 karyotypes were selected for counting, and an additional 5 karyotypes were selected for further detailed analysis, in accordance with the guidelines outlined in the International System for Human Cytogenomic Nomenclature (ISCN 2016).

The abnormalities diagnosed using chromosome karyotype analysis included aneuploidy, deletions, duplications, mosaicism, balanced and unbalanced translocations, inversions, small supernumerary marker chromosomes. However, chromosomal polymorphism was excluded from the diagnosis (relevant cases presented in Supplement Table 3).

### CMA

For each case, 10mL of amniotic fluid was selected. The procedure was carried out in accordance with the manufacturer’s guidelines, as detailed in our previously published work [15]. Annotation was performed using the Genome Reference Consortium Human Build 37 (GRCh37), and the database utilized for data analysis has been detailed in our previous research [15]. The interpretation of CNVs was based on the consensus guidelines provided by the American College of Medical Genetics and Clinical Genome Resource (ACMG-ClinGen). CNVs were divided into five categories: pathogenic, likely pathogenic, variants of uncertain significance (VUS), likely benign and benign. The study primarily focuses on the discussion of pathogenic and likely pathogenic CNVs as well as uniparental disomies (UPDs).

### Pregnancy outcomes and postnatal follow-up

All cases underwent follow-up on pregnancy outcomes via telephone interviews and medical record reviews. For cases with abnormal prenatal diagnostic results who were born, a secondary follow-up was conducted at 3–5 years of age through telephone and in-person interviews to evaluate postnatal health examination outcomes, including systematic physical examinations, organ-specific functional assessments, metabolic/hematological testing, and neurobehavioral development evaluations.

### Statistical processing

Statistical analysis was conducted using statistical software SPSS19.0. The forest plot was generated using R software (version 3.6.0). The count data were statistically described as the number of cases (percentage) [n (%)]. For the statistical analysis, cases of aneuploidy combined CNVs and aneuploidy combined translocation were classified into the aneuploid group. The chi-square (χ²) test or Fisher’s exact test was used to compare the prevalence of abnormal ultrasound markers across different prenatal diagnosis outcomes. Variables with *P* < 0.1 in univariate analysis were selected for multifactorial binary logistic regression and presented through a forest plot, with *P* < 0.05 considered statistically significant.

## Results

### Case profiles

**Fig. 1:**
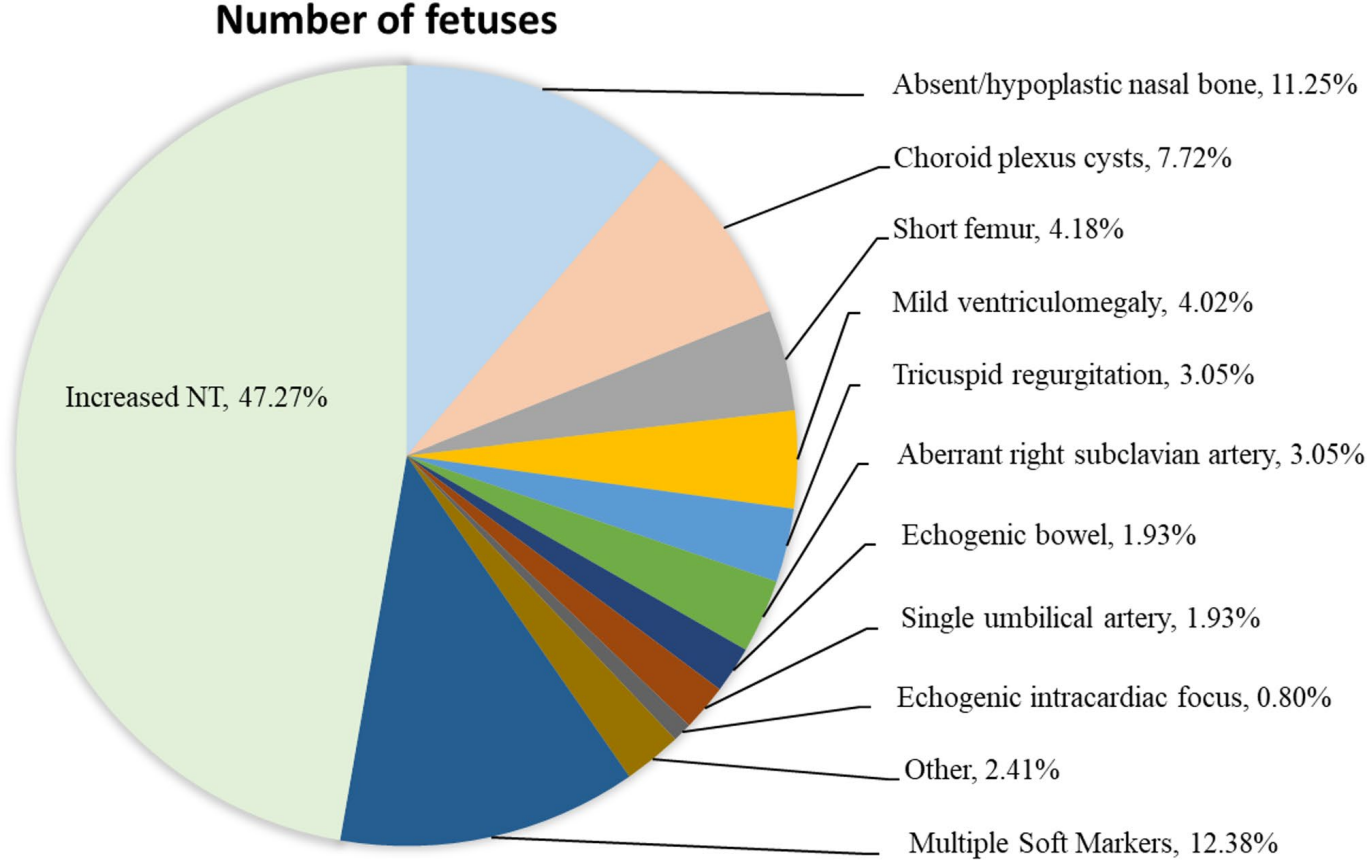
Phenotypic characteristics of 622 prenatal cases

The total number of cases was 622, including 545 cases in the isolated soft marker group and 77 cases in the multiple soft markers group. The average maternal age was 31.6 years, with a range of 19 to 43 years, while the average gestational age for prenatal diagnosis was 20w + 6, ranging from 17w to 28w + 3. Among our cohort, the majority of cases belonged to the increased NT group (294/622, 47.27%), followed by the absent nasal bone group (70/622, 11.25%) and the choroid plexus cyst group (48/622, 7.72%) (shown in Fig. 1; Table 1).

**Table 1:** Prevalence rates of chromosomal abnormalities in different soft marker groups

Ultrasound category	Ultrasound-determined gestational age	Number of fetuses	Chromosomal abnormalities (*n* (%))	Aneuploidies	CNVs	Translocations
Increased NT	First-trimester	294	27(9.18)	20	4	3
Absent nasal bone	First-trimester	70	9(12.86)	8	1	0
Single umbilical artery	First-trimester	12	0(0.00)	0	0	0
Choroid plexus cyst	Second-trimester	48	3(6.25)	1	2	0
Short femur	Second-trimester	26	1(3.85)	1	0	0
Mild ventriculomegaly	Second-trimester	25	0(0.00)	0	0	0
Tricuspid regurgitation	Second-trimester	19	2(10.53)	0	2	0
Aberrant right subclavian artery	Second-trimester	19	1(5.26)	0	0	1
Echogenic bowel	Second-trimester	12	0(0.00)	0	0	0
Echogenic intracardiac focus	Second-trimester	5	3(60.00)	3	0	0
Other	-	15	0(0.00)	0	0	0
Multiple Soft Markers	-	77	25(32.47)	24	0	1
Total	-	622	71(11.41)	57	9	5

### Prevalence of chromosomal abnormalities

**Fig. 2:**
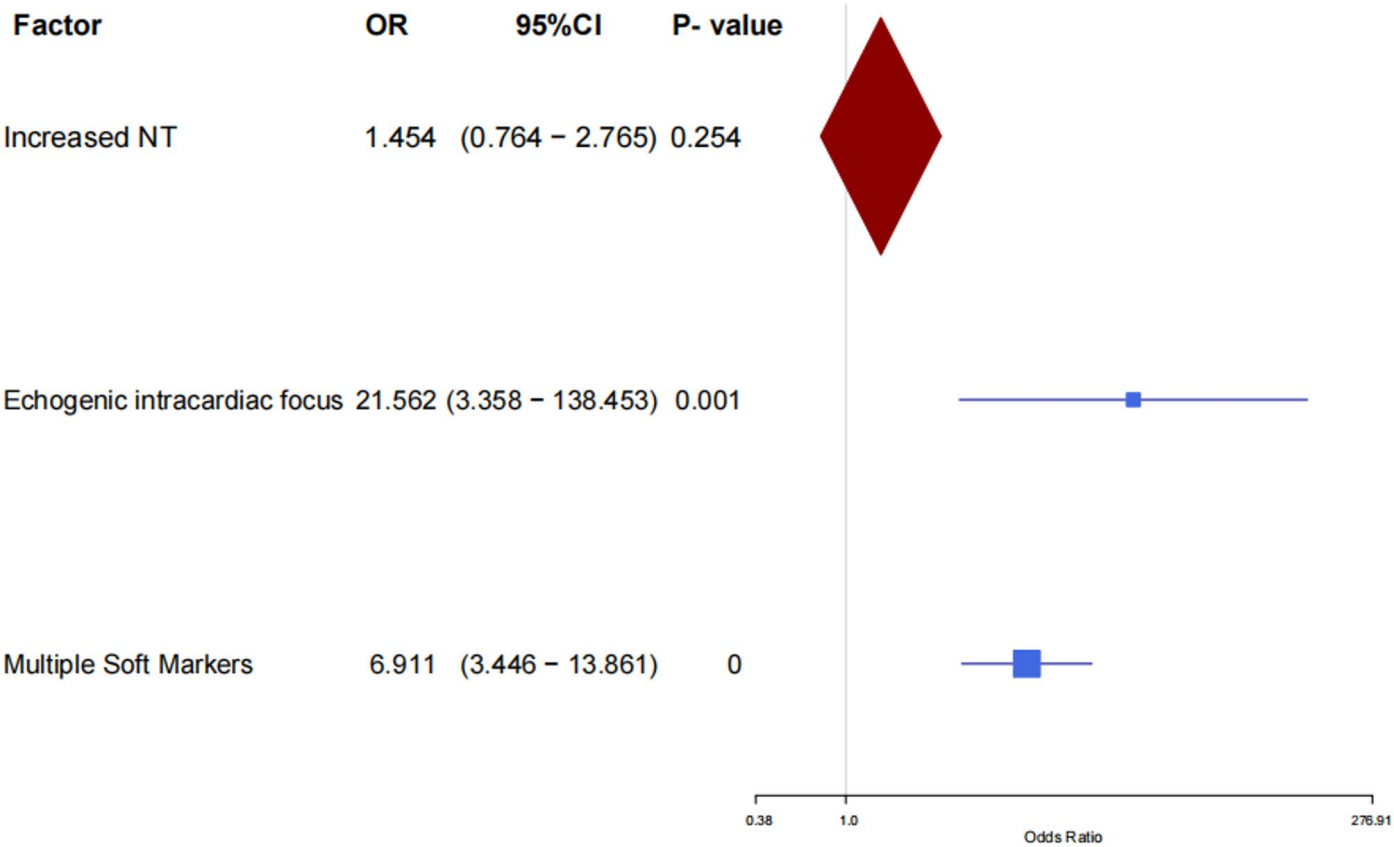
Forest plot of abnormal ultrasound soft markers vs. prenatal diagnosis outcomes

In our cohort, the overall prevalence of chromosomal abnormalities was 11.41% (71/622). The chi-square (χ²) test or Fisher’s exact test revealed that increased NT, echogenic intracardiac focus, and multiple soft markers might be associated with chromosomal abnormalities (shown in Table 2). Further multifactorial binary logistic regression analysis and the forest plot validated that echogenic intracardiac focus, particularly multiple soft markers, demonstrated a highly significant correlation with chromosomal abnormalities (shown in Table 3; Fig. 2). In the echogenic intracardiac focus group, trisomy 21 was identified in 1 case, while complex chromosomal mosaicism accounted for the remaining 2 cases. In the multiple soft markers group, 19 cases of trisomy 21, 5 cases of trisomy 18, 1 case of balanced translocation were identified, with no CNVs detected (shown in Table 4). Statistical analysis using the χ² test revealed a significant difference in the proportions of aneuploidies between isolated and multiple soft marker groups (χ² = 38.519, *P* < 0.001). In contrast, no significant difference was observed in the rate of CNVs between these groups.

**Table 2:** Chi-square test with abnormal ultrasound markers and prenatal diagnoses

Findings on ultrasound	Prenatal diagnostic results		*P*
Increased NT (n (%))	Normal	267(48.46)	0.098^#^
	Aberrant	27(38.03)	
Absent nasal bone (n (%))	Normal	61(11.07)	0.687
	Aberrant	9(12.68)	
Choroid plexus cyst (n (%))	Normal	45(8.17)	0.241
	Aberrant	3(4.23)	
Short femur (n (%))	Normal	25(4.54)	0.344
	Aberrant	1(1.41)	
Mild ventriculomegaly (n (%))	Normal	25(4.54)	0.1
	Aberrant	0(0)	
Tricuspid regurgitation (n (%))	Normal	17(3.09)	1
	Aberrant	2(2.82)	
Aberrant right subclavian artery (n (%))	Normal	18(3.27)	0.712
	Aberrant	1(1.41)	
Echogenic bowel (n (%))	Normal	12(2.18)	0.378
	Aberrant	0(0)	
Single umbilical artery (n (%))	Normal	12(2.18)	0.378
	Aberrant	0(0)	
Echogenic intracardiac focus (n (%))	Normal	2(0.36)	0.012*
	Aberrant	3(4.23)	
Other (n (%))	Normal	15(2.72)	0.239
	Aberrant	0(0)	
Multiple soft markers (n (%))	Normal	52(9.44)	< 0.001***
	Aberrant	25(35.21)	

^#^ *P* < 0.1, *P < 0.05, ** *P* < 0.01, ***P < 0.001

**Table 3:** Binary logistic regression analysis of abnormal ultrasound soft markers and prenatal diagnoses

Related factors	B	SE	Wal	*P*-value	OR	95% CI	
						Lower	Upper
Increased NT	0.374	0.328	1.3	0.254	1.454	0.764	2.765
Echogenic intracardiac focus	3.071	0.949	10.477	0.001**	21.562	3.358	138.453
Multiple Soft Markers	1.933	0.355	29.64	< 0.001***	6.911	3.446	13.861

*SE* Standard error, *OR* odds ratio, *CI* confidence interval; **, *P* < 0.01; ***, *P* < 0.001

### Results of chromosome karyotype analysis

A total of 63 cases of the abnormality were diagnosed by the chromosome karyotype analysis with the prevalence of 10.13% (63/622). The largest number of these abnormalities was 47,XN,+21, followed by 47,XN,+18. Apart from aneuploidies, some special chromosomal abnormalities were diagnosed. There were two cases of complex chromosomal mosaicism, namely mos45,X[4]/47,XYY[38]/46,XY[5] and mos45,X[2]/47,XXX[2]/46,XX[57]. Five cases were diagnosed as balanced translocation. Two additional cases were aneuploidy combined translocation, both of which were 46,XX, rob(13;21)(q10;q10),+21. Meanwhile, one small supernumerary marker chromosome was detected, and further testing was required to identify its origin (shown in Table 4).

**Table 4:** Results of chromosome karyotype analysis

Ultrasound category	Chromosome karyotype analysis results									
	47, XN, + 21	47, XN, + 18	45, XO	47, XXX	47, XXY	47, XYY	mosaicism	translocations	aneuploidy combined translocation	small supernumerary marker chromosomes
Increased NT	11	2	4	1	0	1	0	3	1	0
Absent nasal bone	6	0	0	0	1	0	0	0	1	0
Short femur	1	0	0	0	0	0	0	0	0	0
Choroid plexus cyst	0	1	0	0	0	0	0	0	0	0
Echogenic intracardiac focus	1	0	0	0	0	0	2	0	0	0
Aberrant right subclavian artery	0	0	0	0	0	0	0	1	0	0
Tricuspid regurgitation	0	0	0	0	0	0	0	0	0	1
Multiple soft markers	19	5	0	0	0	0	0	1	0	0
Total	38	8	4	1	1	1	2	5	2	1

### Results of CMA

CMA was performed on 622 prenatal cases with ultrasound soft markers, resulting in the prevalence of 10.45% (65/622). There were 55 cases of aneuploidies, 1 case of the aneuploidy combined pathogenic CNVs, 6 cases of pathogenic CNVs, and 3 cases of likely pathogenic CNVs. The CNVs were associated with duplications of 17q12, 12p13.33p11.23, 16p13.11; deletions of 16p13.12p13.11, 3q29, 16p11.2, 15q11.2 and Xp22.31. The cases diagnosed with pathogenic or likely pathogenic CNVs had ultrasonic phenotypes that included tricuspid regurgitation, increased NT, absent nasal bone, and choroid plexus cyst (shown in Table 5).

**Table 5:** Results of CNVs

	Findings on ultrasound	CMA	Size of CNVs (kb)	Pathogenicity
1	Tricuspid regurgitation	arr[GRCh37]16p13.12p13.11(14780640–16527659)x1	1747	Pathogenic
2	NT 3.8 mm	arr[GRCh37]3q29(195718751–197386180)x1	1667	Pathogenic
3	NT 5.3 mm	arr[GRCh37]16p11.2(29567296–30190029)x1	623	Pathogenic
4	NT 3.4 mm	arr[GRCh37]16p11.2(29351826–30190029)x1	838	Pathogenic
5	NT 3.2 mm	arr[GRCh37]15q11.2(22582283–23060000)x1	478	Pathogenic
6	Absent nasal bone	arr[GRCh37]Xp22.31(6537109–8167604)x1	1630	Pathogenic
7	Absent nasal bone	arr[GRCh37]17q12(36466620–37940921)x3,(21)×3	1470	Pathogenic
8	Tricuspid regurgitation	arr[GRCh37]12p13.33p11.23(173786-27350550)x4	27,177	Likely pathogenic
9	Choroid plexus cyst	arr[GRCh37]16p13.11(15077292–16178545)x3	1100	Likely pathogenic
10	Choroid plexus cyst	arr[GRCh37]16p13.11(14892976–16517413)x4	1620	Likely pathogenic

### Resulting comparison of the chromosome karyotype analysis and CMA

In this study, all cases underwent chromosome karyotype analysis and CMA. In most cases, the results of the two tests were consistent, but there were still some cases with different results (shown in Table 6).

For instance, five balanced translocations (cases 1 ~ 5) and one pseudodiploid (case 6) were missed by CMA. In addition, two cases with CMA results of “arr(21)×3” were supplemented by karyotyping (cases 7 and 8).

However, compared to karyotype analysis, CMA diagnosed 9 cases of pathogenic and likely pathogenic CNVs (cases 9 to 17) and supplemented a case initially diagnosed as aneuploidy by karyotype analysis, revealing the presence of both aneuploidy and pathogenic CNV in this case (case 18). It was noteworthy that case 15 was initially diagnosed as a small supernumerary marker chromosome by karyotype analysis and was identified as arr[GRCh37]12p13.33p11.23(173786-27350550) x4 by CMA.

**Table 6:** Summary of cases with different results from chromosome karyotype analysis and CMA

	Findings on ultrasound	Chromosome karyotype analysis	CMA
1	NT 3.0 mm	46,XX, t(11;19)(q25;p13.1)dn	Normal
2	NT 3.3 mm	46,XY, t(3;12)(q26.2;q13)dn	Normal
3	NT3.5 mm	45,XY, rob(14;15)(q10;q10)	Normal
4	Echogenic intracardiac focus, echogenic bowel	46,XX, t(11;22)(q25;q13.1)	Normal
5	Aberrant right subclavian artery	46,XX, t(10;13)(q21.2;q32)dn	Normal
6	Echogenic intracardiac focus	mos45,X[2]/47,XXX[2]/46,XX[57]	Normal
7	Absent nasal bone	46,XX, rob(13;21)(q10;q10),+21	arr(21)×3
8	NT 3.5 mm	46,XX, rob(13;21)(q10;q10)mat,+21	arr(21)×3
9	Tricuspid regurgitation	Normal	arr[GRCh37]16p13.12p13.11(14780640–16527659)x1
10	NT 3.8 mm	Normal	arr[GRCh37]3q29(195718751–197386180)x1
11	NT 5.3 mm	Normal	arr[GRCh37]16p11.2(29567296–30190029)x1
12	NT 3.4 mm	Normal	arr[GRCh37]16p11.2(29351826–30190029)x1
13	NT 3.2 mm	Normal	arr[GRCh37]15q11.2(22582283–23060000)x1
14	Absent nasal bone	Normal	arr[GRCh37]Xp22.31(6537109–8167604)x1
15	Tricuspid regurgitation	47,XX,+mar	arr[GRCh37]12p13.33p11.23(173786-27350550)x4
16	Choroid plexus cyst	Normal	arr[GRCh37]16p13.11(15077292–16178545)x3
17	Choroid plexus cyst	Normal	arr[grch37]16p13.11(14892976–16517413)x4
18	Absent nasal bone	47,XX,+21	arr[GRCh37]17q12(36466620–37940921)x3,(21)×3

### Results of pregnancy outcomes and postnatal follow-up

**Fig. 3:**
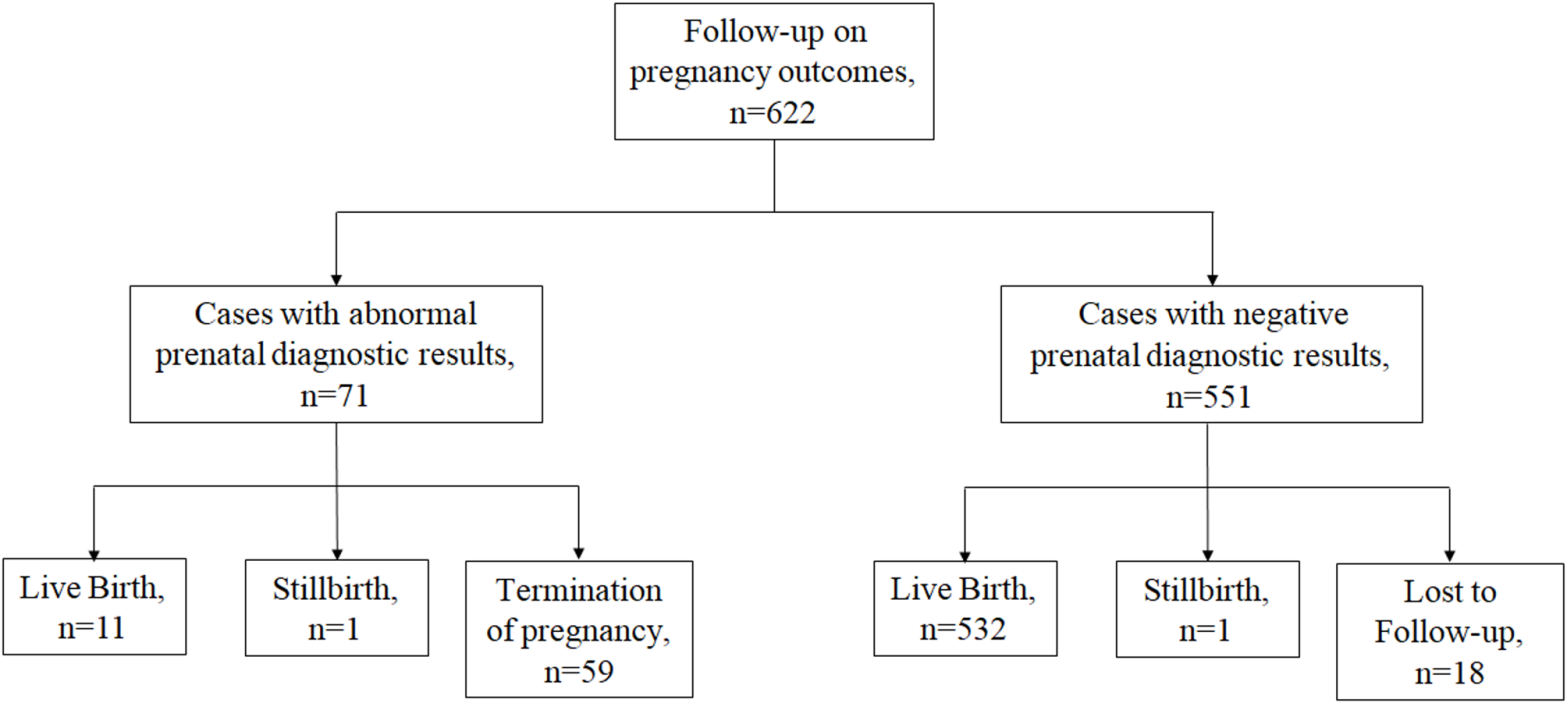
Follow-up of pregnancy outcomes

A follow-up was conducted on the pregnancy outcomes of all 622 cases, confirming the outcomes of 604 cases (excluding 18 lost to follow-up in the prenatal diagnostic negative group). In the group with abnormal prenatal diagnostic results, one case resulted in stillbirth (prenatal ultrasound indicated absent nasal bone), 59 cases underwent termination of pregnancy, and 11 cases resulted in live birth (shown in Fig. 3; Table 7). Secondary follow-up at 3–5 years of age was conducted for the 11 live-born cases, revealing no significant phenotypic abnormalities.

**Table 7:** Live birth cases with abnormal prenatal diagnostic results

	Findings on ultrasound	Chromosome karyotype analysis results	CMA
1	Increased NT	47,XXX	arr(X)×3
2	Increased NT	47,XYY[10]/46,XY[11]	arr(1–22)×2,(X)×1,(Y)×2[0.5]
3	Increased NT	46,XX, t(11;19)(q25;p13.1)dn	Normal
4	Increased NT	47,XYY	arr[GRCh37] Yp11.32q11.223(118551_26192662)x2,Yq11.23(27769668_28799654)x2
5	Increased NT	45,XY, rob(14;15)(q10;q10)	Normal
6	Echogenic intracardiac focus	45,X[2]/47,XXX[2]/46,XX[57]	Normal
7	Echogenic intracardiac focus, echogenic bowel	46,XX, t(11;22)(q25;q13.1)	Normal
8	Absent nasal bone	46,XX	arr[GRCh37] Xp22.31(6537109_8167604)x1
9	Aberrant right subclavian artery	46,XX, t(10;13)(q21.2;q32)dn	Normal
10	Choroid plexus cyst	46,XY	arr[GRCh37] 16p13.11(15077292_16178545)x3
11	Choroid plexus cyst	46,XY	arr[GRCh37] 16p13.11(14892976_16517413)x4

## Discussion

This study focuses on the prenatal diagnosis of fetuses with soft markers using the chromosome karyotype analysis and CMA. The overall prevalence of chromosomal abnormalities was found to be 11.41% (71/622), which was consistent with the previous study performed by Cai et al. (10.17%) [4]. Furthermore, our data demonstrate that the prevalence of chromosomal aneuploidy is significantly higher in the multiple soft marker group compared to the isolated soft marker group. Additionally, through statistical analysis, it was found that the echogenic intracardiac focus may exhibit a correlation with chromosomal abnormalities. However, the number of related cases in this study is limited. Therefore, while this result has heightened our attention to the echogenic intracardiac focus, the current cases are insufficient to support the conclusion regarding its correlation with chromosomal abnormalities, and further validation with a larger cohort remains necessary. For increased NT, existing literature has reported a significant association with chromosomal abnormalities [16]. While a certain correlation was observed in this study, it did not reach statistical significance, indicating the need for further investigation with a larger sample size. Based on the finding of this study and existing literature, special attention should be paid to cases with multiple soft markers, echogenic intracardiac focus, the absent nasal bone and the increased NT [4, 7, 16, 17]. For these cases, the invasive diagnostic genetic testing should not be ignored.

5 cases of balanced translocations were diagnosed by chromosome karyotype analysis, and all of them had normal CMA results. This finding offers valuable reference for future reproductive health strategies of these individuals as they mature. Furthermore, the potential risks associated with balanced translocation should be addressed during prenatal genetic counseling. For example, the Philadelphia chromosome, characterized by *ABL1-BCR* fusion at translocation breakpoints, plays a critical role in leukemogenesis [18, 19]. In addition, a 46,XX, t(4;7)(q27;p22) case with *PCDH10* and *TNRC18* mutations at the translocation breakpoint presented neurodevelopmental delay, facial dysmorphism and high myopia [20]. Therefore, it is essential to assess the inheritance pattern and the family history, and gene sequencing of breakpoints should be considered for fetuses with de novo mutations. In this study, three of the five cases of the balanced translocation underwent parental genetic verification, and all of them were de novo mutations. Among the five cases, only one underwent pregnancy termination, while the remaining four demonstrated normal physical development during the 3–5year follow-up period. Certainly, their physical condition will be monitored continuously.

In the current study, a limited number of pathogenic and likely pathogenic CNVs were identified, notably including two cases of 16p11.2 deletion syndrome and two cases of 16p13.11 duplication. The 16p11.2 deletion syndrome is mainly manifested as intellectual disability, developmental delay, epilepsy, overweight and spinal deformity, while skeletal deformity, cardiovascular deformity and central nervous system deformity are the predominant phenotypes in fetal period [21, 22]. In this study, both cases of 16p11.2 deletion were associated with increased NT, so as to supplement the fetal phenotype of 16p11.2 deletion cases. The 16p13.11 duplication is a neuro susceptible locus, and the clinical phenotype of patients mainly involves neurological diseases, such as schizophrenia [23]. Although few cases of 16p13.11 duplication fetuses have described phenotypes such as echogenic bowel and corpus callosum agenesis, no established consensus on clinical features exists [24, 25]. Notably, both 16p13.11 duplication cases in this study exhibited choroid plexus cysts, suggesting this finding as a potentially significant fetal phenotype. Both cases were delivered without complications, and a 3-year follow-up revealed no abnormal phenotypic features. However, given the 3-year follow-up limitation, delayed-onset manifestations cannot be entirely ruled out; thus, extended follow-up remains essential.

Additionally, CMA analysis revealed 40 chromosomal variations classified as VUS, including 36 CNVs and 4 UPDs (shown in Supplementary Tables 1 and 2). Given that these variations are classified as VUS and the case presented in this article only involves abnormal ultrasound soft markers, we exercise caution in prenatal interpretations and do not definitively categorize them as chromosomal abnormalities. However, for reference, we present the ultrasound phenotype and CMA results, believing that they will contribute to the accumulation of information regarding the pathogenicity of these variants. It is noteworthy that this study detected 4 UPDs. CMA, which combines array-CGH and SNP-array, is not only capable of diagnosing CNVs but also UPDs, and performs well in prenatal diagnosis.

In recent years, prenatal diagnosis has seen advancements with the introduction of the whole exome sequencing (WES) [26, 27]. Nevertheless, prenatal WES, as a phenotype-dependent testing, is not a routine detection method for detecting fetuses with abnormal ultrasound soft markers. According to the expert consensus, cases with abnormal soft markers are considered for WES testing only when increased NT, especially NT > 4 mm or multiple soft markers are present [28]. Therefore, CMA and karyotype analysis remain the main methods for prenatal diagnosis of fetuses with ultrasound soft markers [29]. Karyotype analysis has the advantages of intuitionistic analysis, especially in the diagnosis of aneuploidy, mosaicism, and chromosomal translocation. CMA can effectively compensate for the problem of low resolution of karyotype analysis, and can detect additional microdeletion, microduplication and UPDs. The combined use of these two tests can help reduce the risk of missing pathogenic variants.

## Conclusions

In conclusion, the study underscores the importance for pregnant women who exhibit ultrasound soft markers, particularly those with multiple soft markers, to proactively seek prenatal genetic diagnosis. The synergistic combination of CMA and chromosomal karyotype analysis has proven to be a potent tool in elucidating the underlying genetic factors associated with these soft markers, thereby enabling more informed medical decision-making and tailored care.

## Supplementary Information

Supplementary Material 1.

Supplementary Material 2.

Supplementary Material 3.

## Data Availability

Data will be made available on request.

## Abbreviations

CMA: chromosomal microarray analysis
NT: nuchal translucency
ISUOG: International Society of Ultrasound in Obstetrics and Gynecology
NIPT: non-invasive prenatal testing
ISCN 2016: International System for Human Cytogenomic Nomenclature
GRCh37: Genome Reference Consortium Human Build 37
CNVs: Copy Number Variations
ACMG-ClinGen: American College of Medical Genetics and Clinical Genome Resource
VUS: Variants of uncertain significance
UPDs: uniparental disomies
array-CGH: comparative genomic hybridization
SNP-array: single nucleotide polymorphism array
WES: whole exome sequencing
