# Supplementary material for: Prenatal diagnosis of fetuses with ultrasound soft markers
Source: BMC Pregnancy Childbirth. 2025 Nov 6;25:1168. doi: 10.1186/s12884-025-08238-z (PMC12590621; doi:10.1186/s12884-025-08238-z)
Supplement: Supplementary file 1 — Supplementary Material 1. [file 12884_2025_8238_MOESM1_ESM.docx]

| Supplement Table 1 Results of CMA(VUS) | | | | |
| --- | --- | --- | --- | --- |
|  | Findings on ultrasound | CMA | Size of CNVs (kb) | Pathogenicity |
| 1 | Short femur | arr[GRCh37] 4q35.1q35.2(187019904_187587479)x1 | 568 | VUS |
| 2 | Short femur | arr[GRCh37] 17q11.2(28749120_29516669)x3 | 768 | VUS |
| 3 | Short femur | arr[GRCh37] Yq11.23(26287818_27691424)x0 | 1404 | VUS |
| 4 | Choroid plexus cyst | arr[GRCh37] 1q44(247888675_248660805)x1 | 772 | VUS |
| 5 | Choroid plexus cyst | arr[GRCh37] 5p14.3(18716689_22105896)x1 | 3389 | VUS |
| 6 | Tricuspid regurgitation | arr[GRCh37] 7q35q36.1(146981161_147988322)x1 | 1007 | VUS |
| 7 | Tricuspid regurgitation | arr[GRCh37] 11q24.2(125694048_125899272)x3 | 205 | VUS |
| 8 | Tricuspid regurgitation | arr[GRCh37] 12p11.1(33774226_34835641)x3 | 1061 | VUS |
| 9 | Tricuspid regurgitation | arr[GRCh37] Xp22.31(6455151_8135568)x3 | 1680 | VUS |
| 10 | Echogenic bowel | arr[GRCh37] 4p16.1(8212173_8437267)x1 | 225 | VUS |
|  |  | arr[GRCh37] Xp22.33(1779153_2019878)x3 | 2407 | VUS |
|  |  | arr[GRCh37] Xq26.2(130501596_130948588)x4 | 4470 | VUS |
| 11 | Absent nasal bone | arr[GRCh37] 2q36.3(225946087_226447675)x1 | 502 | VUS |
| 12 | Absent nasal bone | arr[GRCh37] 9q31.3(112369841_112603342)x3 | 233 | VUS |
|  |  | arr[GRCh37] 20q13.33(59145573_60920709)x1 | 17751 | VUS |
| 13 | Absent nasal bone | arr[GRCh37] 11p11.2(43486662_44822546)x3 | 1336 | VUS |
| 14 | Absent nasal bone | arr[GRCh37] 15q11.2(22770421_23082237)x1 | 312 | VUS |
| 15 | Single umbilical artery | arr[GRCh37] 21q21.1(20057677_21699658)x1 | 1642 | VUS |
| 16 | Increased NT | arr[GRCh37] 1q44(247575767_248639486)x3 | 1064 | VUS |
| 17 | Increased NT | arr[GRCh37] 2q13q14.1(110614125_112354279)x1 | 1740 | VUS |
| 18 | Increased NT | arr[GRCh37] 4q35.2(188778244_190957460)x1 | 2179 | VUS |
| 19 | Increased NT | arr[GRCh37] 5q23.2(123384297_124452351)x3 | 1068 | VUS |
| 20 | Increased NT | arr[GRCh37] 5q35.3(178730384_178936592)x3 | 206 | VUS |
|  |  | arr[GRCh37] 8p23.1(8093065_8388820)x1 | 2958 | VUS |
|  |  | arr[GRCh37] 19p12(22342971_23217767) | 8748 | VUS |
| 21 | Increased NT | arr[GRCh37] 6q26(162810362_163053738)x1 | 243 | VUS |
| 22 | Increased NT | arr[GRCh37] 6q26(162643775_163695305)x3 | 1052 | VUS |
| 23 | Increased NT | arr[GRCh37] 6q26(162468123_163334304)x3 | 866 | VUS |
| 24 | Increased NT | arr[GRCh37] 7q35(143515071_144173412)x1 | 658 | VUS |
| 25 | Increased NT | arr[GRCh37] 7p22.1p21.3(6990860_8173622)x3 | 1183 | VUS |
| 26 | Increased NT | arr[GRCh37] 8p22p21.3(19184477_20139818)x1 | 955 | VUS |
|  |  | arr[GRCh37] 10q11.22q11.23(45751537_50114612)x1 | 43631 | VUS |
| 27 | Increased NT | arr[GRCh37] 11p11.2p11.12(48086948_49268340)x3 | 1181 | VUS |
| 28 | Increased NT | arr[GRCh37] 11q14.3(90933796_92321523)x3 | 1388 | VUS |
| 29 | Increased NT | arr[GRCh37] 15q11.2(22770421_23191761)x1 | 421 | VUS |
| 30 | Increased NT | arr[GRCh37] 15q11.2(22582283_23060000)x1 | 478 | VUS |
| 31 | Increased NT | arr[GRCh37] 17q24.2q24.3(68508738_71001040)x1 | 2492 | VUS |
| 32 | Increased NT | arr[GRCh37] Xp11.22(53504222_53702401)x2 | 198 | VUS |
| 33 | Mild ventriculomegaly, tricuspid regurgitation, short femur, reversed A-wave in the ductus venosus, single umbilical artery, umbilical cord cyst | arr[GRCh37] 1q41(223192016_223646826)x1 | 455 | VUS |
| 34 | Increased NT, choroid plexus cyst | arr[GRCh37] 1q23.2q23.3(159686992_160870226)x1 | 1183 | VUS |
| 35 | Single umbilical artery, echogenic intracardiac focus | arr[GRCh37] 7q35(143521291_144173151)x1 | 652 | VUS |
| 36 | Absent nasal bone, short femur | arr[GRCh37] 15q11.2(22770421_23282798)x1 | 512 | VUS |
| 37 | Choroid plexus cyst | arr[GRCh37] 6q16.2q22.1(99679232_117534585)x2 hmz | 17855 | VUS |
| 38 | Increased NT | arr[GRCh37] 1q25.3q31.2(181768984_192554617)x2 hmz | 10786 | VUS |
| 39 | Increased NT | arr[GRCh37] 10q21.1q21.3(55139541_68395570) hmz | 13256 | VUS |
|  |  | arr[GRCh37] 15q24.1q25.3(73423044_86340630)hmz | 129176 | VUS |
| 40 | Increased NT | arr[GRCh37] Xp11.3p11.1(44434259_58227320) hmz | 13793 | VUS |
|  |  | arr[GRCh37] Xq11.1q21.1(62036670_76856021) hmz | 148194 | VUS |
| VUS: Variants of uncertain significance | | |  |  |
