# Supplementary material for: Prenatal diagnosis of fetuses with ultrasound soft markers
Source: BMC Pregnancy Childbirth. 2025 Nov 6;25:1168. doi: 10.1186/s12884-025-08238-z (PMC12590621; doi:10.1186/s12884-025-08238-z)
Supplement: Supplementary file 2 — Supplementary Material 2. [file 12884_2025_8238_MOESM2_ESM.docx]

Supplement Table 2 CNVs/UPDs classified as VUS in different soft marker groups

| Ultrasound category | Number of fetuses | Variations (n (%)) | UPDs | CNVs |  |
| --- | --- | --- | --- | --- | --- |
|  |  |  |  |  |  |
| Increased NT | 294 | 20(6.80) | 3 | 17 |  |
| Absent nasal bone | 70 | 4(5.71) | 0 | 4 |  |
| Choroid plexus cyst | 48 | 3(6.25) | 1 | 2 |  |
| Short femur | 26 | 3(11.54) | 0 | 3 |  |
| Mild ventriculomegaly | 25 | 0(0.00) | 0 | 0 |  |
| Tricuspid regurgitation | 19 | 4(21.05) | 0 | 4 |  |
| Aberrant right subclavian artery | 19 | 0(0.00) | 0 | 0 |  |
| Echogenic bowel | 12 | 1(8.33) | 0 | 1 |  |
| Single umbilical artery | 12 | 1(8.33) | 0 | 1 |  |
| Echogenic intracardiac focus | 5 | 0(0.00) | 0 | 0 |  |
| Other | 15 | 0(0.00) | 0 | 0 |  |
| Multiple Soft Markers | 77 | 4(5.19) | 0 | 4 |  |
| Total | 622 | 40(6.43) | 4 | 36 |  |
| UPDs: Uniparental disomies; VUS Variants of uncertain significance | | |  |  |  |
