# Supplementary material for: Prenatal diagnosis of fetuses with ultrasound soft markers
Source: BMC Pregnancy Childbirth. 2025 Nov 6;25:1168. doi: 10.1186/s12884-025-08238-z (PMC12590621; doi:10.1186/s12884-025-08238-z)
Supplement: Supplementary file 3 — Supplementary Material 3. [file 12884_2025_8238_MOESM3_ESM.docx]

| Supplement table 3 Cases of chromosomal polymorphism | | | |
| --- | --- | --- | --- |
|  | Findings on ultrasound | Chromosome karyotype analysis results | CMA |
| 1 | NT 3.0mm | 46,XX,13pss | Normal |
| 2 | NT 3.0mm | 46,XY,22ps- | Normal |
| 3 | NT 3.1mm | 46,XY,9qh+pat | Normal |
| 4 | NT 3.3mm | 46,XY,21cenh+mat | Normal |
| 5 | Absent nasal bone | 46,XY,14pss | Normal |
| 6 | Tricuspid regurgitation | 46,XY,inv(9)(p12q13) | Normal |
| 7 | Echogenic bowel | 46,XX,inv(9)(p12q13) | Normal |
| 8 | Mild ventriculomegaly | 46,XY,22pss | Normal |
| 9 | Mild ventriculomegaly | 46,XY,9qh+mat | Normal |
| 10 | Echogenic bowel | 46,XY,qh+pat | Normal |
